# Major errors in published *Salmonella* risk assessment model

**DOI:** 10.1017/S0950268822000796

**Published:** 2022-05-02

**Authors:** Francisco J. Zagmutt, Régis Pouillot

**Affiliations:** 1 EpiX Analytics, Fort Collins, USA; 2 Independent consultant, Rabat, Morocco

Dear Prof. Noah,

The Sampedro *et al*. [1] publication addresses an important issue in *Salmonella* control. It is very relevant today, given the renewed efforts by the US Department of Agriculture (USDA) to reduce *Salmonella* illnesses attributable to poultry (https://www.usda.gov/media/press-releases/2021/10/19/usda-launches-new-effort-reduce-salmonella-illnesses-linked-poultry). However, we have found significant errors in the modelling methods that will affect the validity of the manuscript.

We do not attempt to provide an exhaustive review of the manuscript, so we will focus only on two key issues that will significantly affect the authors' findings, as they relate to the implementation and integration of the dose-response models used to predict salmonellosis resulting from exposure to the pathogen:

1. Using incorrect dose-response parameters and equations:
2. On table #3 of the paper, the reported *β* parameters of the Beta-Poisson dose-response model for *Salmonella enterica* subsp. *enterica* ser. Anatum (*S*. Anatum) and *S.* Typhimurium are 291 002 and 1 301, respectively. These values were re-parameterized from the following N50 values: 37 100 for *S.* Anatum (http://qmrawiki.canr.msu.edu/index.php/Salmonella_anatum:_Dose_Response_Models) and 49.8 for *S.* Typhimurium (http://qmrawiki.canr.msu.edu/index.php/Salmonella_nontyphoid:_Dose_Response_Models)
3. However, applying the reported parameters in table #3 in the Beta-Poisson dose-response model, we get an N50 of 2 282 541 cells for *S*. Anatum and 33 997 cells for *S*. Typhimurium. These numbers clearly don't match the N50 values that were used to derive the beta parameters.
4. The inconsistency results from using an incorrect formula for N50. The correct formula is *N*50 = *β*  (2^1/*α*^ − 1), as reported in Haas *et al*. [2] [pp. 274, eqn. #8.19]. Solving for the reported parameters allowed us to infer that the incorrect formula *N*50 = *β*/(2^1/*α*^ − 1) was used. Notice (1) that this formula substitutes the product with a division; (2) that this error is probably linked to a typographical error in the first edition of Haas *et al*. [3] [pp. 268, eqn #7.20]. Assuming that the Beta-Poisson approximation holds for these values, the correct parameters should be *α* = 0.318, *β* = 4 730 for *S*. Anatum and *α* = 0.21, *β* = 1.906 for *S*. Typhimurium.
5. The Beta-Poisson equations in Table #3 also have two errors:
  - o The Beta-Poisson approximation should be *P*(inf|*D*) = 1 − (1 + *D*/*β*)^−*α*^, instead of *P*(inf|*D*) = 1 − (1 − *D/β*)^*α*^
  - o The equation from the Teunis *et al*. [4] model was written in the article as *P*(*ill*|*inf*, *D*) = 1 − (1 − *ηD*^−*ρ*^); the correct formula is *P*(*ill*|*inf*, *D*) = 1 − (1 + *ηD*)^−*ρ*^
6. The mistake in the parameters results in artificially flat dose-response functions that would be almost linear at the dose ranges modelled. For example, using the *S.* Anatum dose-response with the reported parameters above for an ingested dose of 37 100 cells leads to a probability of illness estimated as 1–(1 + (37 100/291 002))^−0.318^ = 3.7% whereas this should be exactly 50% if the parameters were correct (as the N50 represents the dose resulting in 50% chance of infection). Likewise, the mistake in the parameters for *S*. Typhimurium resulted in a model that requires a dose over 650 times greater to infect 50% of exposed individuals.
7. The mistake in the equations will considerably alter the predictions and will result in mathematical errors such as the fractional power of a negative number.
8. The combination of the issues above would result in serious biases and would impede the proper evaluation of enumeration-based criteria for *Salmonella*.
9. Incorrectly integrating the exposure and DR equations
10. The supplementary material in Sampedro *et al.* [1] shows a prediction of illnesses ranging from very few to 50 M. Evidently this scale of predictions is not feasible as it does not match surveillance data or other previously published *Salmonella* risk assessments.
11. It is common to use adjustment factors to match the median/mean quantitative microbial risk assessment (QMRA) model predictions to surveillance data. However, the variance of the predictions in this manuscript is too broad and inconsistent with other QMRA predictions.
12. Typically when we see a result like this in a QMRA, it stems from not performing the integration of the dose (D) and dose-response *P(Illness|D)* correctly to arrive to the probability of illness given any contamination i.e. *P*(Illness|Contamination) = ∫_>0_^∞^ *P*(*Illness*|*D*) *f*(*D*)*dD*, where *f*(*D*) is the probability distribution of the ingested doses in the population of interest. The authors probably considered one iteration of their Monte Carlo simulation (representing the risk of salmonellosis for an expected dose) as the mean risk for the whole population. This is a fairly ubiquitous error when using spreadsheet add-ons to perform risk assessments, as done in this article. Unfortunately, this creates a major distortion in the prediction of illnesses.

In summary, although we did not review the spreadsheet model used in this article, we have found significant errors that will affect the study's findings. We urge the authors to correct these errors while also reviewing the spreadsheet model for any other possible errors that might compromise their findings. Beyond the issues that we have described, the model relies on some assumptions that may not have been tested for robustness. For example, the dose-response models for selected *Salmonella* serovars were assumed to predict illnesses from a broad group of other *Salmonella* serovars present in turkey. The robustness of the model to these and other assumptions warrants further investigation.

This article could provide relevant evidence that regulators and industry can use to consider possible strategies to reduce *Salmonella* illnesses attributable to turkey. For this and future publications, we urge the authors to include the spreadsheet model as supplementary material to ensure transparency and reproducibility of their findings.

## References

[1] Sampedro F (2018) Developing a risk management framework to improve public health outcomes by enumerating Salmonella in ground Turkey. Epidemiology and Infection 147, e69. 30520390 10.1017/S095026881800328X PMC6518596

[2] Haas CN, Rose JB and Gerba CP (2014) Quantitative Microbial Risk Assessment, 2nd Edn. Hoboken, New Jersey: Wiley.

[3] Haas CN, Rose JB and Gerba CP (1999) Quantitative Microbial Risk Assessment, 1st Edn. Hoboken, New Jersey: Wiley.

[4] Teunis PFM (2010) Dose-response modeling of Salmonella using outbreak data. International Journal of Food Microbiology 144, 243–249. 21036411 10.1016/j.ijfoodmicro.2010.09.026

